# Memory-Associated Immediate Early Genes: Roles in Synaptic Function, Memory Processes, and Neurological Diseases

**DOI:** 10.1007/s12035-025-05203-x

**Published:** 2025-07-26

**Authors:** Zafar U. Khan, Marta Carretero-Rey, Cristina A. Muñoz de León-López, Irene Navarro-Lobato

**Affiliations:** 1 Laboratory of Neurobiology, Centro de Investigaciones Medico Sanitarias (CIMES), University of Malaga, University of Malaga, Campus Teatinos s/n, Malaga, 29010 Spain; 2 Department of Medicine, Faculty of Medicine, University of Malaga, Campus Teatinos S/N, Malaga, 29010 Spain; 3 Centro de Investigación Biomédica en Red, Enfermedades Neurodegenerativas (CIBERNED), Institute of Health Carlos III, Madrid, Spain

**Keywords:** Immediate early genes, AP1, CFos, CJun, Npas4, Zif268 (Egr1), Homer1a, Arc (Arg3.1), BDNF, Narp, Memory, Synaptic plasticity, LTP and LTD, Aging and neurological diseases

## Abstract

The expression of immediate early genes (IEGs) in the brain is rapidly upregulated during learning or in response to an event. This upregulation often correlates with neuronal activity in interconnected brain regions that form circuits associated with memory processing and formation. IEGs function either as transcription factors regulating gene expression or as effector proteins primarily involved in synaptic activities. AP-1 is a dimer composed of members of the Fos, Jun, ATF, and Maf transcription factor families. Its composition is a critical determinant of the expression of specific gene sets. AP-1 regulates a broad range of genes and is activated by various stimuli, including stress, drugs, learning, and exposure to new events. Other IEG transcription factors, such as Zif268 (Egr-1) and Npas4, regulate the transcription of genes essential for structural and synaptic plasticity. Conversely, effector proteins like Homer1a, Arc (Arg3.1), BDNF, and Narp contribute to AMPA receptor trafficking, its internalization, and both Hebbian and non-Hebbian forms of synaptic plasticity. Both types of IEGs play a critical role in memory and synaptic plasticity. Alterations in their function are associated with cognitive dysfunction in aging, as well as various neurological and psychiatric diseases. This review provides an overview of the current understanding of both types of IEGs in the regulation of different forms of synaptic plasticity, their contributions to memory functions, and their roles in aging and brain diseases.

## Introduction

The transcription of immediate early genes (IEGs) is initiated within minutes of neuronal activation. This swift response enables neurons to adapt to experiences and changing environmental conditions, making IEGs essential for neuronal functions such as synaptic plasticity, memory formation, and stress responses. IEGs in the brain act as transcription factors to modulate gene expression or as effector proteins primarily involved in synaptic functions [1]. Table 1 highlights only those implicated in memory and synaptic plasticity. Once translated and post-translationally modified in the cytoplasm, IEGs that function as transcription factors are transported into the nucleus, where they bind to specific sites in the promoter and enhancer regions of target genes to promote gene transcription. In contrast, IEGs acting as effector proteins directly engage in biological activities to modulate synaptic functions. The activation of IEGs immediately after an experience or event has been shown to be a crucial step in the formation of long-lasting memory [1, 2], and their activation is critical for the induction of synaptic plasticity [3–5]. Moreover, the rapid activation characteristic of IEGs has served as a valuable tool for tagging active neuronal ensembles, allowing researchers to track neurons selectively engaged during specific behavioral or sensory experiences and to study activity-regulated neuronal populations and memory engrams [6, 7].

**Table 1:** Immediate early genes in the brain associated with memory function and synaptic plasticity

IEGs	Family	Function	Reference
**Transcription factors**			
ATF3	bZIP	Regulates gene expression in response to various cellular stress signals, and is involved in synaptic plasticity and memory	[8, 9]
c-Fos	Fos	Regulates gene expression in response to a variety of extracellular stimuli, and is implicated in synaptic plasticity and memory consolidation	[2, 10]
c-Jun	Jun	Regulates gene expression in response to various cellular stimuli, and plays a role in synaptic plasticity, learning, and memory processes	[11, 12]
Egr3	EGR	Regulates gene expression to facilitate neuronal plasticity, memory formation, and immune system regulation	[13, 14]
FosB	Fos	Regulates gene expression in response to a variety of stimuli, and is involved in neuronal plasticity, memory formation, and addiction	[15, 16]
Fra-1	Fos	Functions as transcriptional regulator in response to a several stimuli, and is essential for development, stress response, and synaptic functions	[17, 18]
JunB	Jun	Regulates gene expression in cellular processes such as stress adaptation, synaptic plasticity, and memory formation	[19, 20]
Npas4	bHLH-PAS	Regulates neuronal activity-dependent gene transcription critical for plasticity and inhibitory synapse formation	[21, 22]
Nurr1(NR4A2)	NR4A	Essential for dopaminergic neurons development and maintenance, synaptic plasticity, and memory formation	[23, 24]
Nurr77 (NR4A1)	NR4A	Regulates gene transcription implicated in growth and survival of dopaminergic neurons, synaptic plasticity, and long-term memory	[25, 26]
SRF	MADS-box	Regulates gene expression involved in cell growth, proliferation, synaptic plasticity, and learning	[27, 28]
Zif268 (Egr1)	EGR	Regulates gene expression critical for learning and memory and synaptic plasticity	[29, 30]
**Effector proteins**			
Homer1a	Homer protein	Regulates synaptic plasticity, homeostatic scaling, and endocytosis of AMPA receptors	[31, 32]
Arc (Arg3.1)	N/A	Plays a role in synaptic plasticity, AMPA receptor endocytosis, and memory consolidation	[33, 34]
BDNF	Neurotrophin	Plays a role in brain development, synaptic regulation, and memory processes	[35–37]
Cox2	COX	Plays a role in inflammation and injury, and is involved memory and synaptic plasticity	[38, 39]
Gadd45b	Gadd45	Regulates neuronal plasticity and is involved in inflammation and immune response	[40, 41]
Gadd45γ	Gadd45	Plays a role in DNA repair, cell cycle regulation, stress response, and memory	[42, 43]
Narp	Pentraxin	Plays a role in synaptic function and plasticity and is involved in clustering of AMPA receptors	[44–46]
Neuritin (CPG15)	Neuritin	Involved in the regulation of synaptic plasticity, memory, and neuronal development	[47, 48]
Plk2 (Snk)	PLK	Regulates memory processes, long-term potentiation, and homeostatic plasticity	[49, 50]
RGS2	RGS	Regulates neuronal signaling essential for learning, memory and synaptic plasticity	[51, 52]
tPA	Serine protease	Implicated in neuronal processes such as synaptic plasticity, learning, and memory	[53, 54]
VGF	Secreted neuropeptides	Involved in synaptic transmission, neuroplasticity, and learning and memory	[55, 56]

The selection of IEGs is based on behavioral studies in live animals that demonstrate their involvement in memory. *ATF3* activating transcription factor 3, *c-Fos* cellular FBJ murine osteosarcoma viral oncogene homolog, *c-Jun* cellular Jun proto-oncogene, *Egr3* early growth response 3, *FosB* FBJ murine osteosarcoma viral oncogene homolog B, *Fra-1* Fos-related antigen 1, *JunB *Jun B proto-oncogene, *Npas4* neuronal PAS domain protein 4, *Nurr1* nuclear receptor–related 1 protein, *Nurr77* nuclear receptor–related 77 protein, *SRF* serum response factor, *Zif268* zinc finger protein 268, *Homer1a* Homer scaffold protein 1a, *Arc* activity-regulated cytoskeleton–associated protein, *BDNF* brain-derived neurotrophic factor, *Cox2* cyclooxygenase-2, *Gadd45b* growth arrest and DNA damage–inducible 45 beta, *Gadd45γ* growth arrest and DNA damage–inducible 45 gamma, *Narp* neuronal activity–regulated pentraxin, *Plk2* polo-like kinase 2, *RGS2* regulator of G-protein signaling 2, *tPA* tissue plasminogen activator, *VGF* VGF nerve growth factor inducible

Although numerous IEGs associated with memory function and synaptic plasticity are listed in the table under both categories, not all are well-studied. Therefore, we have focused on those with relatively extensive literature: c-Fos, c-Jun, Npas4, and Zif268 as transcription factors, and Homer1a, Arc, BDNF, and Narp as effector proteins.

## IEGs of Transcription Factors

Transcription factors play a critical role in regulating gene expression, acting as molecular switches that determine when and how genes are activated or suppressed in response to stimuli. Among the diverse array of transcription factors, IEGs represent a unique category distinguished by their rapid expression.

### The IEG Family of AP-1 Dimer

**Fig. 1:**
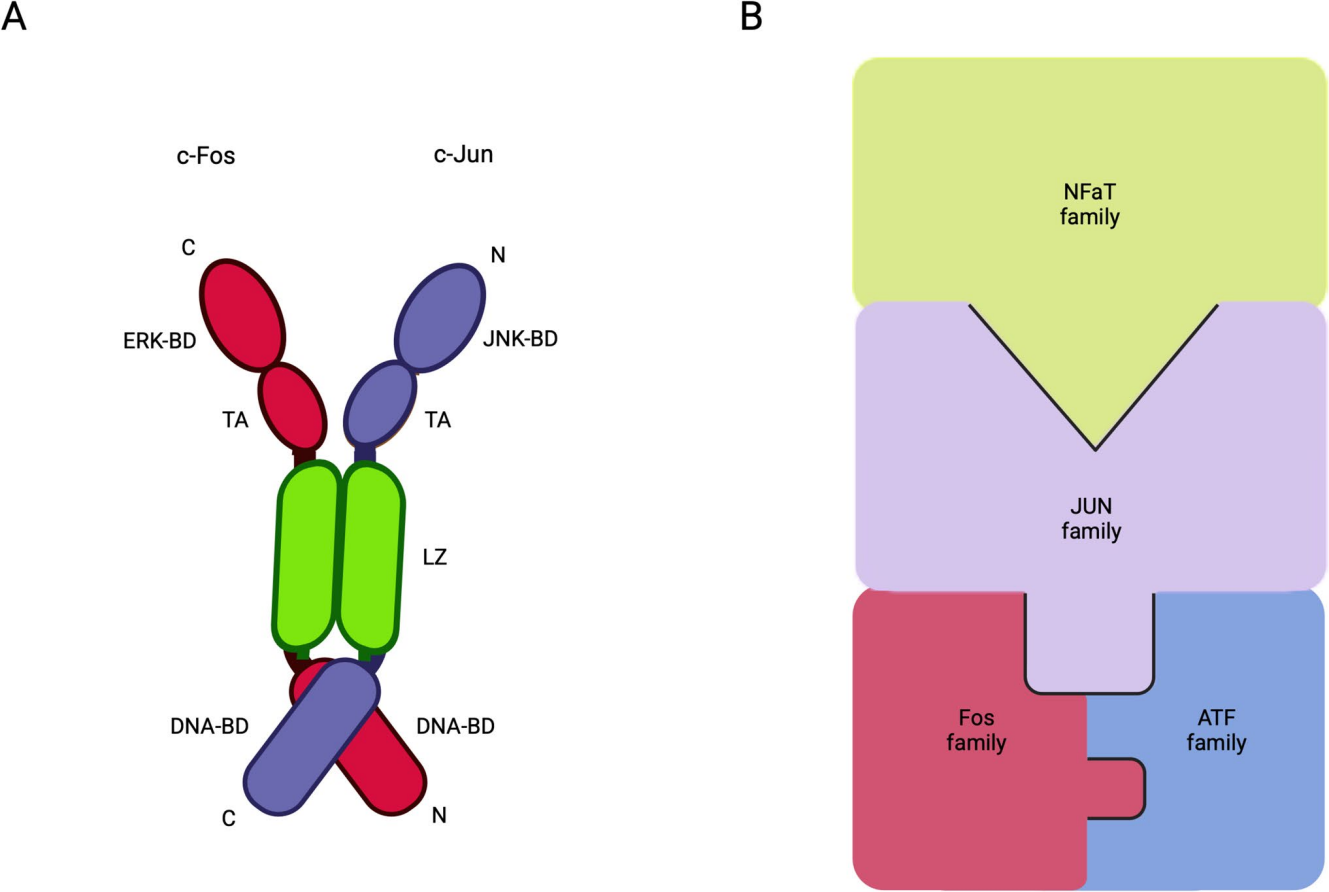
AP-1 transcription factor dimers.** A** A dimer composed of c-Jun from the Jun family and c-Fos from the Fos family. Each component contains a DNA-binding domain (DNA-BD), a leucine zipper (LZ) region, a transcription-activating module (TA), and specific binding domains: an ERK-binding domain (ERK-BD) in c-Fos and a JNK-binding domain (JNK-BD) in c-Jun. **B** Representation of dimer formation among members of the AP-1 family. Jun family members interact with all other AP-1 families, while Fos family members form dimers exclusively with members of the Jun and ATF families. N, NH2-terminal; C, COOH-terminal

Activator protein-1 (AP-1) consists of dimers composed of members from the Fos, Jun, ATF (activating transcription factor), and Maf families [57]. The Fos family, which includes c-Fos, FosB, Fra-1, and Fra-2, does not form homodimers but can heterodimerize with members of the Jun family (Fig. 1A). In contrast, the Jun family, which includes c-Jun, JunB, and JunD, can homodimerize or heterodimerize with other Jun or Fos members to form transcriptionally active complexes for the regulation of gene expression [57, 58]. The ATF family members associated with AP-1 are primarily ATF2, ATF3, and ATF7, which heterodimerize with Jun family members [59], while the Maf family members—MafA, MafB, and c-Maf—heterodimerize with Fos and Jun family members [60]. Additionally, JDP2, which is a member of the JDP (Jun dimerization protein) family, heterodimerizes with Jun and ATF family members [61]. Therefore, the possible combinations of dimer interactions are numerous, as summarized in Fig. 1B. However, the function of all AP-1 combinations is still unknown. It has been shown that some AP-1 combinations may have overlapping functions and target the same genes [18].

**Fig. 2:**
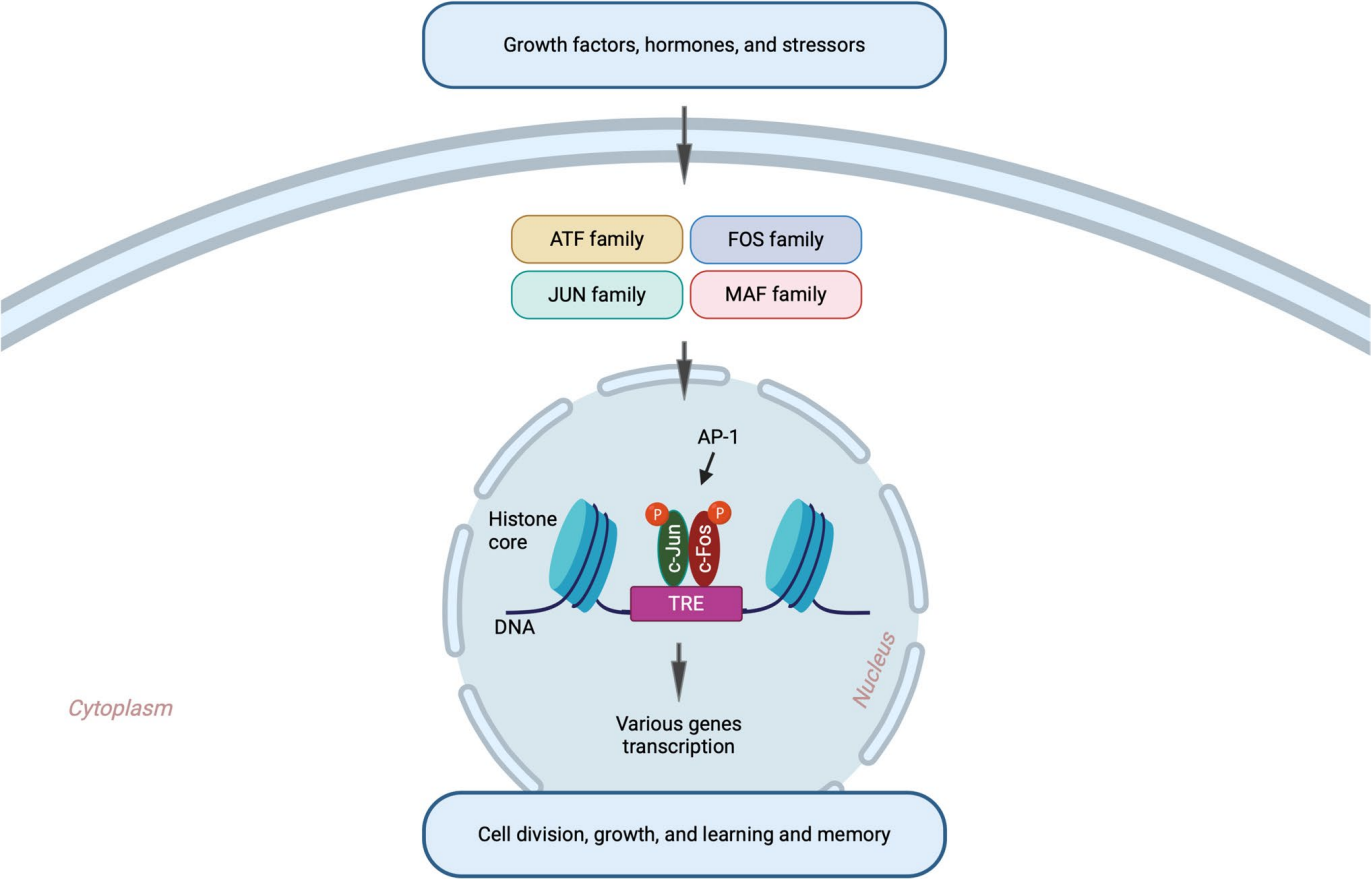
AP-1 responds to diverse extracellular stimuli. Upon activation, the AP-1 dimer complex binds to TRE sequences located in the promoter regions of target genes within the genome to regulate the transcription of genes involved in various biological functions, including learning and memory

AP-1 is activated by protein kinases, after which the activated AP-1 binds to AP-1 binding sites containing the TRE sequence (5′-TGAG/CTCA-3′) in the promoter regions of target genes through its DNA-binding domain to regulate gene expression [62, 63]. The composition of AP-1 is an important functional determinant of both the affinity for DNA binding and the extent of transcriptional activation [58, 62, 64]. AP-1 is involved in many cellular and physiological functions, and it integrates myriads of extracellular signals (Fig. 2). However, cFos and c-Jun are not c-Jun arethe primary AP-1 components involved in memory-related processes, especially in the hippocampus and other brain regions critical for learning, synaptic plasticity, and long-term memory formation. Therefore, in the following sections, we will elaborate on both of them.

#### c-Fos

c-Fos is one of the first transcription factors shown to be induced in an activity-dependent manner [65, 66]. It contains a kinase-binding domain, a transactivation domain, a DNA-binding domain, and a leucine zipper (Fig. 1A). The leucine zipper domain facilitates dimerization with Jun family members to form the AP-1 complex, which functions as a transcription factor [62, 67]. c-Fos acts as both an activator and repressor of gene transcription depending on the interacting partners in the formation of the AP-1 complex. For example, dimerization with c-Jun leads to activation, while binding with ATF causes repression [68, 69].

##### c-Fos in Synaptic Plasticity

Induction of long-term potentiation (LTP) by theta-burst stimulation in the cerebellum induces NMDA receptor-dependent c-Fos expression and CREB phosphorylation [70]. Similarly, electrical stimulation of the sciatic nerve also induces LTP and increases c-Fos expression [71]. In the perforant path, both high-frequency and tetanic stimulation that induce LTP also lead to increased c-Fos expression [72, 73]. In line with these findings, reduced c-Fos expression in rats and c-Fos deletion in mice cause impairments in LTP [10, 74], indicating that c-Fos expression may be a crucial step in the induction of LTP.

Along the same lines as LTP, low-frequency stimulation that induces long-term depression (LTD) in the hippocampus is also associated with elevated c-Fos expression, suggesting that increased c-Fos expression is required for both LTP and LTD. Furthermore, intracerebral application of c-Fos antisense prevents LTD and inhibits the increase in c-Fos expression [75]. In addition, long-term desensitization of cerebellar Purkinje cells, which underlies LTD, induces a synergistic increase in the expression of c-Fos and JunB [76].

##### c-Fos in Memory

c-Fos is widely distributed throughout the brain, and an increase in neuronal activity induces an upregulation of this protein [77, 78]. Studies have shown that c-Fos expression following behavioral training specifically correlates with learning and memory performance [79–84], and the activation of cellular ensembles following memory retrieval [85–87]. Exposure to a novel object results in a significantly greater increase in c-Fos levels in certain brain structures compared to the presentation of familiar objects in rats [88]. The novelty of an object is associated with increased c-Fos activity in two brain areas, i.e., the perirhinal cortex and the visual association area Te2, with no change in the hippocampus, whereas visual associative recognition is linked to changes in c-Fos levels in the hippocampus, but not in the perirhinal cortex [89]. A distributed change in c-Fos expression levels was observed in the rodent brain after exposure to spatial novelty [90]. Daily exposure to acoustic stimuli results in a decrease in c-Fos expression in the auditory pathway of juvenile rats compared to acute stimulation [91]. Exposure to a novel taste increases the expression of c-Fos in the limbic and cortical brain structures of mice [88].

Blocking c-Fos activation in the hippocampus impairs long-term memory associated with water-maze learning, radial-arm maze performance, contextual fear learning, and socially transmitted food preferences [92–95]. Brain infusion of c-Fos antisense blocks the acquisition and extinction of conditioned taste aversion [96]. c-Fos knockout mice exhibited impairments in spatial long-term memory and showed a reduction in LTP at hippocampal CA3-CA1 synapses [10]. Altogether, these findings highlight the importance of c-Fos in various types of memory, in addition to recognition memory.

Despite a broad understanding of c-Fos expression during learning and memory, its precise biological role in the brain remains unknown. It is uncertain whether c-Fos expression is merely a response to neuronal activity within interconnected memory circuits, or if its activation triggers downstream mechanisms within those circuits to facilitate memory processing, formation, and storage. Nonetheless, it has been shown that c-Fos is essential for the formation of memory engrams [97]. Blocking of c-Fos-positive engram neurons linked to aversive olfactory memory inhibits memory recall, whereas activating them induces the associated behavior [97]. c-Fos is also necessary for the maintenance of inhibitory avoidance related memory for long-term storage [95]. Thus, evidence suggests that c-Fos might play a role in memory processes beyond merely responding to neuronal activity.

##### c-Fos in Aging and Neurological Diseases

c-Fos expression levels decrease with age [98], and cognitive decline correlates with altered c-Fos expression in the hippocampus [99]. In contrast to aging, an increase in c-Fos expression has been observed in Alzheimer’s disease [100–102], schizophrenia [103], pain [104, 105], epilepsy [106, 107], anxiety [108–110], and stress [111–113]. The decrease in c-Fos expression with aging and its increased expression in Alzheimer’s disease and other neurological disorders suggest that the AP-1 dimer, formed by c-Fos, may be critical in the upregulation of gene transcription involved in pathways detrimental to neuronal function, such as inflammation and reactive oxygen species, ultimately leading to neuronal dysfunction and even neuronal death.

#### c-Jun

**Fig. 3:**
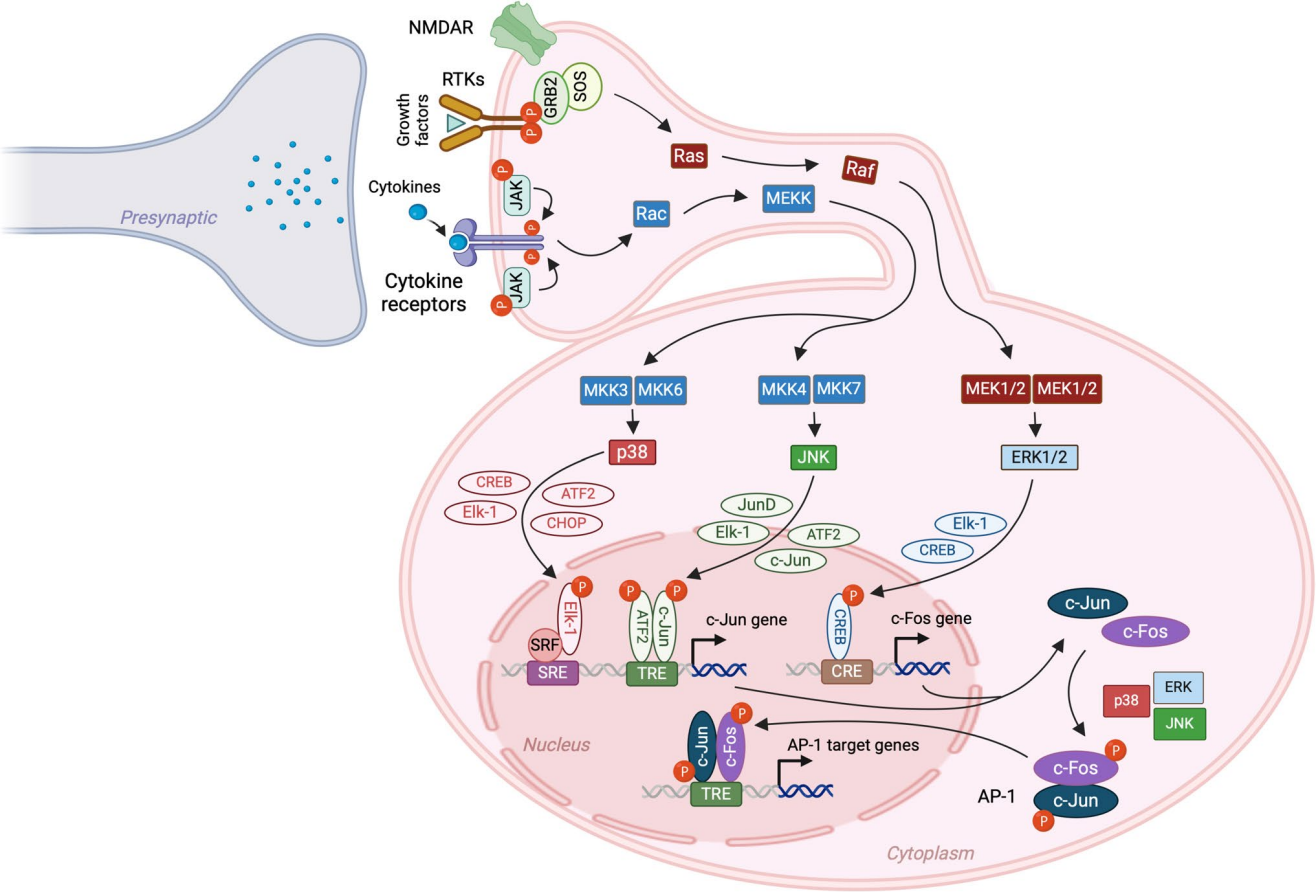
MAP kinase signaling pathways leading to gene transcription mediated by the AP-1 dimer of c-Jun and c-Fos. External stimuli, such as growth factors or inflammatory cytokines, activate mitogen-activated protein kinases (MAPKs) — ERK, p38, and JNK — through the RAS-Raf-MEK or Rac-MEKK-MKK pathways. Once activated, ERK, p38, and JNK translocate to the nucleus, where they phosphorylate transcription factors (shown next to the arrows), which bind as monomers or, in some cases, as dimers to specific binding sites (such as SRE, TRE, or CRE) in the promoter region of the gene to promote transcription of c-Fos and c-Jun. In the cytoplasm, the newly transcribed c-Fos and c-Jun mRNAs are translated into proteins, which are subsequently transported to the nucleus to form the AP-1 dimer and promote transcription of a variety of genes. NMDAR, N-methyl-d-aspartate receptor; RTKs, receptor tyrosine kinases; JAK, Janus kinase; GRB2, growth factor receptor-bound protein 2; SOS, son of sevenless; MEK, mitogen-activated protein kinase kinase; MEKK, mitogen-activated protein kinase kinase kinase; MKK, mitogen-activated protein kinase kinase; p38, p38 mitogen-activated protein kinase; ERK, extracellular signal-regulated kinase; JNK, c-Jun N-terminal kinase; CREB, cAMP response element-binding protein; CHOP, C/EBP homologous protein; ATF2, activating transcription factor 2; Elk-1, ETS like-1; SRF, serum response factor; SRE, serum response element; TRE, TPA response element; CRE, cAMP response element. (Note: All the transcription factors described are nuclear proteins. In some instances, they are depicted outside the nucleus due to space limitations.)

Similar to c-Fos, c-Jun is composed of a kinase-binding domain, a transactivation domain, and a leucine zipper (Fig. 1A). The regulation of gene transcription by c-Jun depends on post-translational modifications at three key regions: (i) in the N-terminal region and phosphorylation of the Ser63 and Ser73 residues, as well as the Thr91 and Thr93 residues [114, 115]; (ii) near the C-terminal region, dephosphorylation of the Thr239 residue [115]; and (iii) in the C-terminal region, acetylation of the Lys268, Lys271, and Lys273 residues [116]. These post-translational modifications are largely regulated by the MAPK family of serine/threonine kinases [117]. c-Jun interacts with Fos family members and other Jun family members to form the AP-1 complex [57, 58] (Fig. 3). In addition, c-Jun can heterodimerize with other transcription factors, such as members of the ATF family [118], and other basic leucine zipper-containing transcription factors, including CBP, MyoD, NFAT, and c-Rel [119]. Therefore, c-Jun is regarded as a major component of the AP-1 complex.

It has been shown that stimulation by growth factors activates extracellular signal–regulated kinases 1/2 (ERK1/2), which phosphorylate c-Jun and the Fos family members Fra-1 and Fra-2 [120]. ERK-mediated phosphorylation of c-Jun near its DNA-binding domain inhibits its ability to bind DNA; however, phosphorylation of Fra-1 and Fra-2 by ERK enhances their DNA-binding activity in conjunction with c-Jun [120, 121]. In contrast to growth factors, the induction of the AP-1 complex by pro-inflammatory cytokines and genotoxic stress is primarily mediated by the c-Jun N-terminal kinase (JNK) and p38 MAPK pathways [122] (Fig. 3). Once act activated, JNKs translocate to the nucleus and phosphorylate c-Jun at serine (Ser63 and Ser73) and threonine (Thr91 and Thr93) residues within the transactivation domain, enhancing its ability to activate transcription when homodimerized or heterodimerized with c-Fos [121, 123]. JNK also phosphorylate ATF2 at Thr69 and Thr71, promoting its transcriptional activity after heterodimerization with c-Jun [124, 125].

##### c-Jun in Synaptic Plasticity

During LTP and LTD, c-Jun expression is strongly increased [126, 127], and it has been shown to contribute to long-term facilitation [12]. c-Jun mutant mice, in which the active phosphorylation sites at serine 63 and serine 73 were replaced with alanine, showed impaired LTP at hippocampal CA3-CA1 synapses; however, LTD was normal [128].

##### c-Jun in Memory

c-Jun expression is associated with memory [11, 129]. Spatial novelty, the acquisition of spatial memory, and the formation of olfactory memory induce c-Jun expression [90, 129, 130]. Spinal cord stimulation through painful sensory stimuli upregulates c-Jun [131, 132]. In mice, training on a bar-pressing task induces c-Jun and c-Fos expression in the hippocampus [133]. In line to these findings, blocking c-Jun expression in the hippocampus impairs discrimination performance in rats [126].

##### c-Jun in Aging and Neurological Diseases

The basal and inducible levels, as well as the DNA-binding activity of c-Jun, are altered in the aged brain [134–137]. Increased expression of c-Jun has been observed in seizures [138, 139], cerebral ischemia and stroke [140, 141], addiction [142, 143], stress [144, 145], and pain [146, 147], and Alzheimer’s disease [102, 148]. Additionally, phosphorylated c-Jun is present in the brains of patients with Alzheimer’s disease [149], and reducing the activity of its phosphorylation enzyme, c-Jun N-terminal kinase, is seen as a potential therapeutic strategy for Alzheimer’s disease [150, 151].

### Zif268

Zif268 (also known as Egr-1, Krox 24, and NGFI-A) belongs to the early growth response family of zinc finger transcription factors that bind to GC-rich response elements in the promoter region to regulate gene expression [152]. The Zif268 protein contains three zinc finger sequences in its DNA-binding domain, which enable it to regulate the transcription of other genes [153, 154]. Additionally, Zif268 can modulate epigenetic programming via DNA methylation and histone post-translational modifications to influence downstream gene regulation [155]. Zif268 responds to various stimuli [77, 156, 157] and is associated with memory processing [158–160]. In particular, hippocampal Zif268 increases in an NMDAR-dependent manner after LTP induction, playing a crucial role in the maintenance of the late phase of hippocampal LTP and the consolidation of spatial memory [161–163].

**Fig. 4:**
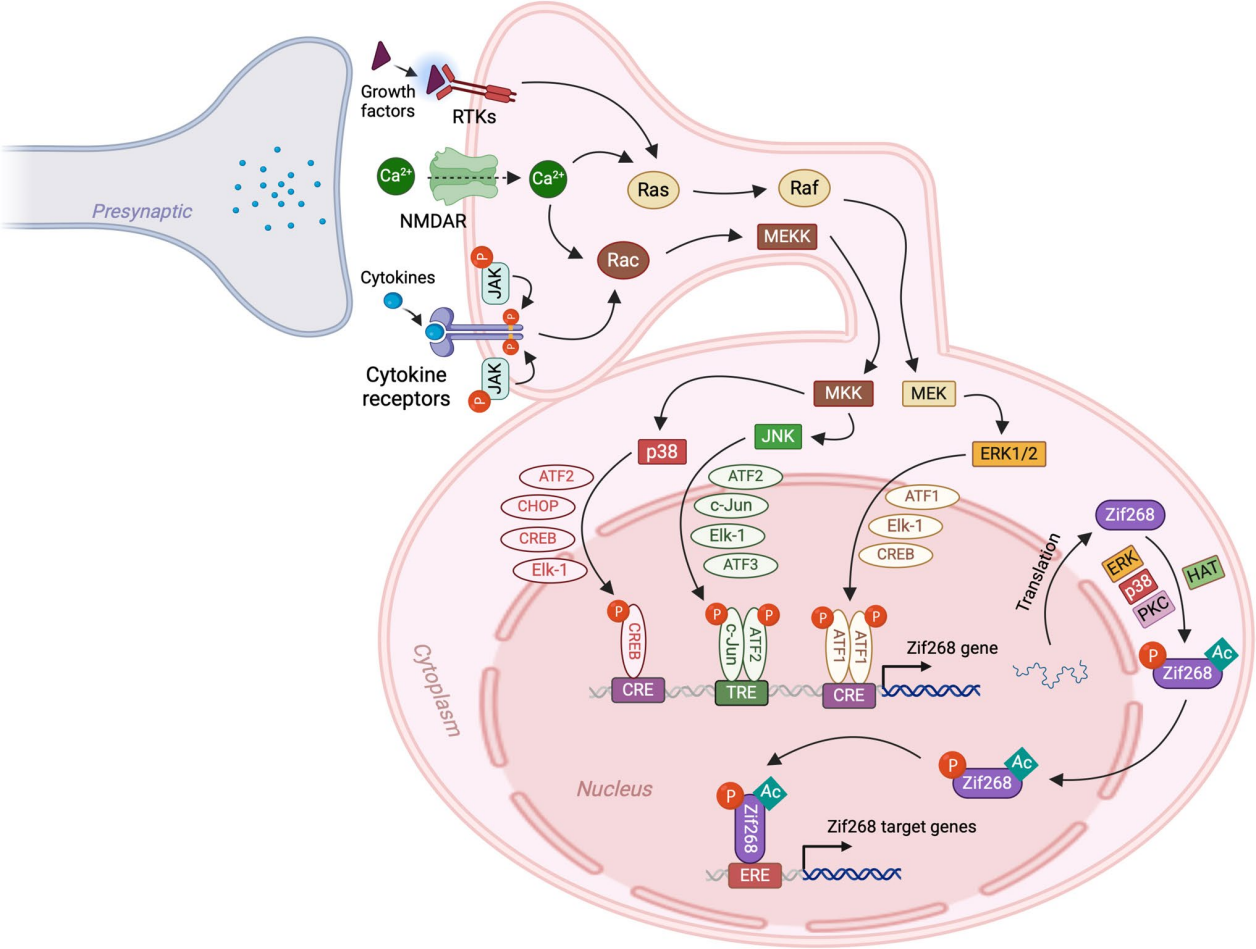
MAP kinase signaling pathways in Zif268 gene transcription and Zif268-mediated gene transcription. After activation by external stimuli, mitogen-activated protein kinases (MAPKs) — ERK, p38, and JNK — translocate to the nucleus, where they phosphorylate transcription factors (shown next to the arrows). These transcription factors bind as monomers or, in some cases, as dimers to specific binding sites (such as CRE, TRE, or ERE) in the promoter region of the Zif268 gene to regulate its transcription. The newly transcribed Zif268 mRNAs are translated into proteins in the cytoplasm, which are then transported to the nucleus. Zif268 proteins are subsequently phosphorylated by MAPKs or acetylated by HAT to facilitate the transcription of various target genes. RTKs, receptor tyrosine kinases; NMDAR, N-methyl-d-aspartate receptor; JAK, Janus kinase; MEKK, mitogen-activated protein kinase kinase kinase; MKK, mitogen-activated protein kinase kinase; MEK, mitogen-activated protein kinase kinase; p38, p38 mitogen-activated protein kinase; ERK, extracellular signal-regulated kinase; JNK, c-Jun N-terminal kinase; CREB, cAMP response element-binding protein; CHOP, C/EBP homologous protein; ATF1, activating transcription factor 1; ATF2, activating transcription factor 2; Elk-1, ETS like-1; ATF3, activating transcription factor 3; PKC, protein kinase C; HAT, histone acetyltransferase; CRE, cAMP response element; TRE, TPA response element; ERE, estrogen response element. (Note: All the transcription factors described are nuclear proteins. In some instances, they are depicted outside the nucleus due to space limitations.)

The promoter region of Zif268 contains several key regulatory sites, including two CREs, six SREs, an Sp1-like motif, CCAATT sequences, AP-1-like motifs, AP-2-like binding sequences, and a GSG box [153, 164–167]. As a result, several signaling pathways and kinases can regulate the transcription of Zif268 (Fig. 4). Multiple receptors, such as glutamate, dopamine, adrenergic, and opiate receptors, which engage distinct signaling cascades, regulate the expression of Zif268 mRNA [168]. Additionally, an elevation in Ca^2^⁺ has been shown to mediate the expression of Zif268 [169]. The known kinases and transcription factors that interact with the promoter region include Elk-1, which binds to SRE sites; CREB, which binds to CRE sites; the Fos and Jun family dimerized complex, which binds to AP-1 sites; and Egr family proteins, which bind to the GSG box [119, 168].

#### Zif268 in Synaptic Plasticity

An induction of LTP increases Zif268 mRNA levels in granule cell neurons, and an NMDA receptor antagonist or blockage of LTP interrupts this increase [170]. Additionally, the induction of LTP in the dentate gyrus of the hippocampus and spinal neurons are associated with the rapid and robust transcription of Zif268 [171, 172]. The mRNA expression of Zif268 is regulated through the MAPK/MEK pathway and is dependent on the ERK1/2, Elk1, and CREB signaling cascade [29, 173]. Zif268 expression is associated with the persistence of LTP rather than its induction [127, 171, 174]. Hippocampal early LTP remains intact in *Zif268* knockout mice; however, LTP was not observed 24–48 h after tetanic stimulation [158, 175]. Thus, Zif268 appears to be specifically required for the expression of late-phase LTP. In addition to LTP, Zif268 is also upregulated in response to DHPG-mediated induction of LTD; however, loss of Zif268 did not result in LTD deficits [176].

#### Zif268 in Memory

Both learning and memory recall lead to an increase in Zif268 gene expression [156, 177]. A knockout of the Zif268 gene in mice induces deficiencies in spatial learning and spatial recognition memory [156, 178] and impairs the formation of stable hippocampal place cell representations of novel environments [163]. In contrast, spatial memory impairment induced by NMDA receptor inhibition attenuates Zif268 expression [179]. Furthermore, the expression of Zif268 is increased in corticolimbic brain structures after the reactivation of consolidated fear memory [180, 181]. Regulation of histone methylation at the Zif268 promoter facilitates contextual fear memory [182], and object exploration triggers rapid phosphorylation, acetylation, and methylation of histones at the Zif268 promoter in the hippocampus and prefrontal cortex [183]. Zif268 in the hippocampus is necessary for the reconsolidation of object recognition memory, but not for its retrieval or storage [30]. Knockdown of the Zif268 gene abolishes the active memory trace during reconsolidation of object recognition memory, and the administration of DNA antisense to Zif268 mRNA impairs object recognition memory [30, 184]. Blocking epigenetic marks at the Zif268 promoter impairs recognition memory, while enhancing these marks through intensive training or transgenic intervention promotes recognition memory [30]. Additionally, Zif268 expression is also critical for fear conditioning [159, 185], eyeblink conditioning [186], and aversive memory [187].

#### Zif268 in Aging and Neurological Diseases

Age-related impaired memory recall following trace fear conditioning is associated with an increase in Zif268 protein accumulation and a decrease in proteolytic activity [188]. However, reduced Zif268 expression has been observed in Alzheimer’s disease mice [189, 190]. It has been shown that Zif268 mediates the phosphorylation of tau protein in the brain [191], and knockout of the Zif268 gene reduces the expression of amyloid precursor protein (APP), while overexpression of APP in the brains of mice increases the expression of Zif268 [192, 193]. Additionally, Zif268 is a transcriptional activator of the BACE1 gene, which is implicated in the synthesis of APP and the deposition of amyloid beta (Aβ) in the brain [194]. Inhibition of Zif268 in the hippocampus alleviates Alzheimer pathology and improves cognition in Alzheimer’s disease mice [195]. Zif268 is one of the genes associated with schizophrenia, and it resides in region 69 of the 108 loci linked to this condition [196]. Zif268 expression is downregulated in schizophrenia [197], whereas it is upregulated in stress [198, 199], ischemic stroke [200], and epilepsy [201]. Similarly, initial cocaine exposure induces the expression of Zif268 in the striatum through dopamine signaling [202, 203]. Zif268 is necessary for the development of cocaine conditioned place preference, and its expression is reactivated upon exposure to drug-related environments, even after prolonged abstinence [204, 205].

### Npas4

**Fig. 5:**
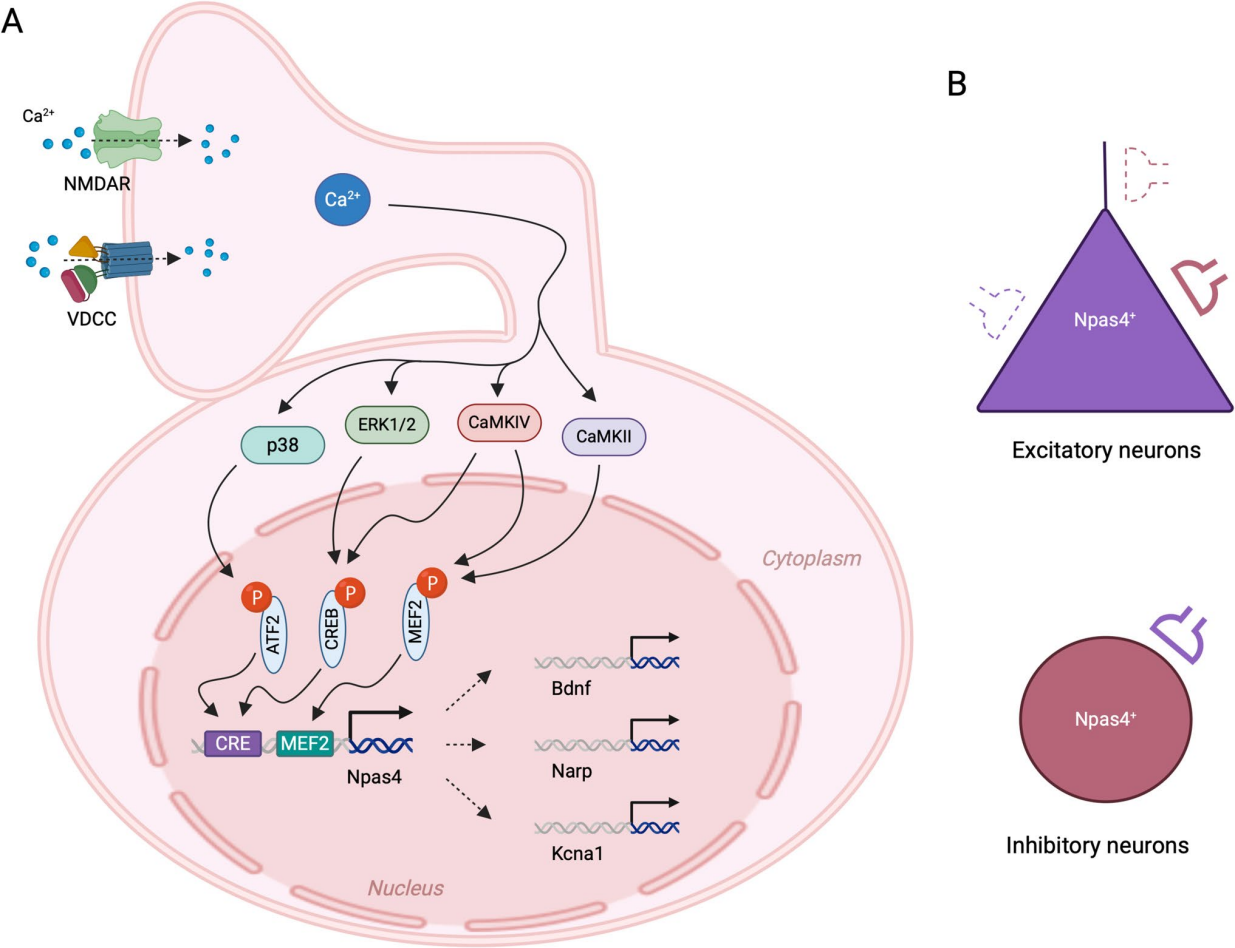
Calcium-mediated Npas4 gene transcription and its role in synapse formation. **A** An increase in intracellular Ca^2^⁺ levels in neurons activates MAP kinases (p38 and ERK1/2) and CaM kinases (CaMKII and CaMKIV) through distinct signaling pathways. These activated kinases translocate to the nucleus and phosphorylate transcription factors — ATF2, CREB, and MEF2 — at specific binding sites, such as CRE and MEF2, in the promoter region of the *Npas4* gene, driving its transcription. After translation of the newly transcribed *Npas4* mRNA in the cytoplasm, the Npas4 proteins are transported back to the nucleus, where they promote the transcription of various genes, including *Bdnf*, *Narp*, and *Kcna1*. **B** Npas4 expression in the brain increases the number of inhibitory synapses (originating from inhibitory neurons) onto pyramidal (excitatory) neurons. NMDAR, N-methyl-d-aspartate receptor; VDCC, voltage-dependent calcium channel; p38, p38 mitogen-activated protein kinase; ERK, extracellular signal-regulated kinase; CaMKII, calcium/calmodulin-dependent protein kinase II; CaMKIV, calcium/calmodulin-dependent protein kinase IV; ATF2, activating transcription factor 2; CREB, cAMP response element-binding protein; CRE, cAMP response element; MEF2, myocyte enhancer factor 2; BDNF, brain-derived neurotrophic factor; Narp, neuronal activity–dependent pentraxin; *Kcna1*, gene encoding voltage-gated potassium channel Kv1.1

Neuronal PAS domain-containing protein 4 (Npas4) is a member of the PAS family, characterized by a conserved basic-helix-loop-helix motif and PAS domain, and it plays a key role in forming inhibitory synapses [206, 207]. Npas4 facilitates the transcription of other genes (Fig. 5A). Reduced expression of Npas4 decreases the number of inhibitory synapses formed onto hippocampal pyramidal neurons, while overexpression leads to an increase in these inhibitory synapses [208] (Fig. 5B, C). Additionally, selective deletion of the Npas4 gene in somatostatin-expressing inhibitory neurons results in a lower density of excitatory synapses onto these neurons [209]. Thus, Npas4 controls the excitatory and inhibitory balance through the formation of both excitatory and inhibitory synapses to maintain neurocircuit homeostasis, essential for information processing and memory formation [208, 209]. Knockout of Npas4 also attenuates the neural activity-dependent induction of numerous genes and abolishes the recruitment of RNA Polymerase II to the promoter and enhancer regions of both c-Fos and brain-derived neurotrophic factor (BDNF), genes crucial for memory formation [210].

#### Npas4 in Plasticity

Npas4 regulates the expression of a large number of genes, including those involved in synaptic plasticity [210]. However, a direct role for Npas4 in LTP or LTD has not been described yet. Alternatively, Npas4 influences the plasticity of synaptic connections that underlie experience-dependent learning and memory by promoting synapse formation and regulating inhibitory transmission [207]. Experience-dependent expression of Npas4 also supports plasticity in the adult visual cortex [211]. In the hippocampus, Npas4 increases the number of synapses onto CA1 pyramidal neurons made by cholecystokinin-expressing basket (CCKB) GABAergic neurons [212]. Furthermore, sensory experience-driven Npas4 expression enhances depolarization-induced suppression of inhibition—a form of cannabinoid-mediated plasticity—at CCKB neuron synapses [212]. In contrast, somatostatin-expressing GABAergic neuron-specific knockout of Npas4 in CA1 alters synaptic transmission in these neurons, increases activity in pyramidal neurons, and reduces anxiety behavior [213]. Similarly, Npas4 deletion in somatostatin-expressing GABAergic neurons in the primary motor cortex disrupts motor learning–induced spine reorganization onto excitatory pyramidal neurons, impairing motor learning [214]. Additionally, behaviorally driven expression of Npas4 in the hippocampus coordinates the redistribution of inhibitory synapses onto CA1 pyramidal neurons, increasing inhibitory synapse numbers on the cell body while decreasing them on the apical dendrites [215].

#### Npas4 in Memory

The absence or downregulation of Npas4 induces deficits in memory and cognitive flexibility [216, 217]. Npas4 expression is required for both new and reactivated auditory fear memory [218] and is associated with the formation of long-term contextual and contextual fear memory [219]. Contextual learning induces Npas4 expression specifically in the CA3 area of the hippocampus, and selective deletion of Npas4 impairs contextual memory formation, while restoring Npas4 in the CA3 area reverses this deficit [210]. Npas4 regulates the structure and strength of synaptic connections between hippocampal mossy fibers and CA3 pyramidal neurons, which are critical to contextual memory encoding, by restricting the number of synaptic contacts [22]. Deletion of the Npas4 gene impairs contextual memory formation and prevents learning-induced modifications in these synaptic connections [22]. Furthermore, biphasic expression during contextual fear conditioning showed that Npas4 can also function as an inducible memory suppressor of highly salient aversive experiences [220].

#### Npas4 in Aging and Neurological Diseases

A decrease in Npas4 expression in the hippocampus is linked to memory impairment in aging [221]. Downregulation of Npas4 in the brain is also associated with major depressive disorder [222] and stress [223–225]. Npas4 deficiency combined with prenatal stress impairs social recognition in mice [226], while changes in Npas4 expression associated with early-life maternal separation stress affect social behavior in adult female mice [227]. Furthermore, exposure to transient stress leads to increased methylation in the Npas4 promoter region and a significant decrease in Npas4 expression [228, 229]. In contrast, Npas4 expression is upregulated in focal cerebral ischemia [230, 231], and Npas4-deficient mice display altered anxiety and social behavior following focal cortical stroke [232]. Additionally, Npas4 is implicated in addiction, where it plays a critical role in regulating neural circuits associated with addictive behaviors [233–236].

## IEGs of Effector Proteins

### Homer1a

**Fig. 6:**
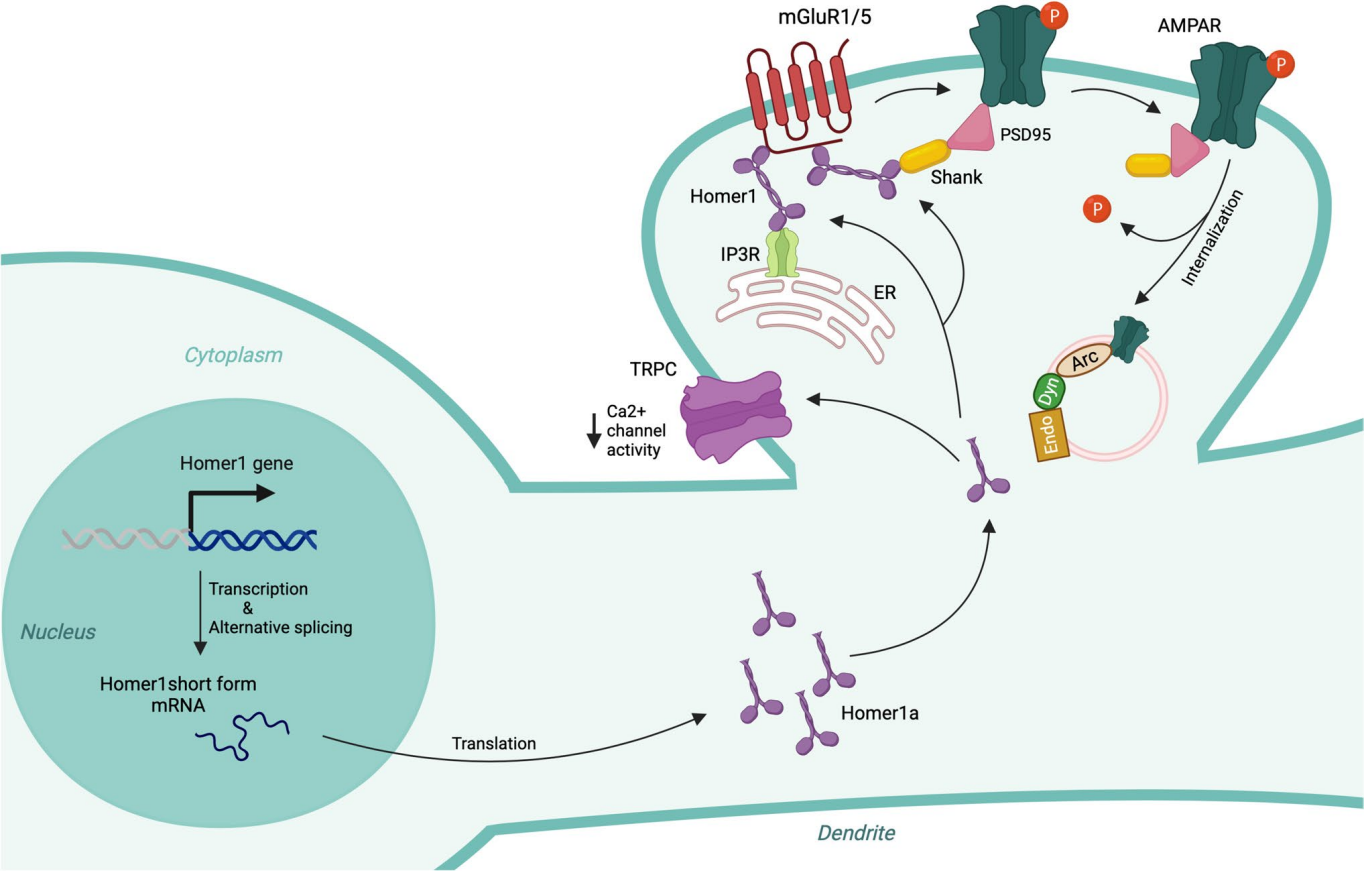
Homer1a in synaptic functions. Neuronal activity upregulates Homer1 gene transcription, followed by alternative splicing to produce the short isoform Homer1a. Homer1a mRNA is translated into protein in the cytoplasm, where it participates in synaptic functions. Under basal conditions, mGluR1/5, channels, and other interacting proteins are cross-linked by the long isoform Homer1. However, upon neuronal activity, the long isoform is replaced by Homer1a. This replacement disrupts mGluR1/5-mediated Ca^2^⁺ release from the endoplasmic reticulum (ER) by uncoupling mGluR1/5 from the IP3 receptor (IP3R) and interrupts the interaction between mGluR1/5 and AMPA receptors (AMPARs) via shank. Binding of Homer1a to mGluR1/5 triggers dephosphorylation and internalization of synaptic surface AMPARs. Additionally, Homer1a blocks the Ca^2^⁺ channel activity of transient receptor potential canonical (TRPC) channels. mGluR1/5, metabotropic glutamate receptor 1/5; PSD95, postsynaptic density protein 95; Arc, activity-regulated cytoskeleton-associated protein; Dyn, dynamin; Endo, endophilin

Homer1a, a shorter isoform of Homer1, is an activity-induced IEG and a member of the Homer scaffold protein family [237, 238]. Both the short (Homer1a) and long (Homer1b and Homer1c) isoforms of Homer1 are generated through alternative splicing of the Homer1 gene, also known as Vesl-1. While Homer1a is upregulated during convulsive seizures and induction of LTP, the long isoforms (Homer1b and Homer1c) are constitutively expressed [237, 239]. The short and long isoforms share the same N-terminal binding domain, also called the EVH1 domain, which is involved in mediating protein–protein interactions [240]. Upon recruitment to the postsynaptic density, Homer1a disrupts interactions between the long Homer1 isoforms and target proteins, which include metabotropic glutamate receptors (mGluRs), inositol trisphosphate receptors, ryanodine receptors, the Shank family of postsynaptic scaffold proteins, and C-type transient receptor potential channels (TRPCs) [238, 241] (Fig. 6). Thus, Homer1a acts as a negative regulator of the activity of the long Homer1 isoforms, modulating synaptic structure and function [238, 242]. By inhibiting the actions of the long isoforms, Homer1a regulates Ca^2^⁺ homeostasis [243–246] and the function of mGluRs [247, 248]. Neuronal activation-mediated expression of Homer1a has been shown to cause dissociation of the mGluR/IP3 receptor complex, leading to a reduced mGluR-mediated calcium response [238].

Homer1a expression is upregulated following experience-dependent activation of sensory and hippocampal neurons [237, 249–253]. Overexpression of Homer1a inhibits dendritic spine morphogenesis and reduces the size of PSD-95 and NMDA receptor clusters, as well as synaptic surface levels of AMPA receptors [254]. Conversely, Homer1a deletion decreases C-terminal phosphorylation at the Y876 site of the GluA2 subunit of AMPA receptors, resulting in enhanced synaptic surface retention of GluA2-containing AMPA receptors and increased synaptic transmission [255]. These effects are reversed when Homer1a expression is normalized [255].

**Fig. 7:**
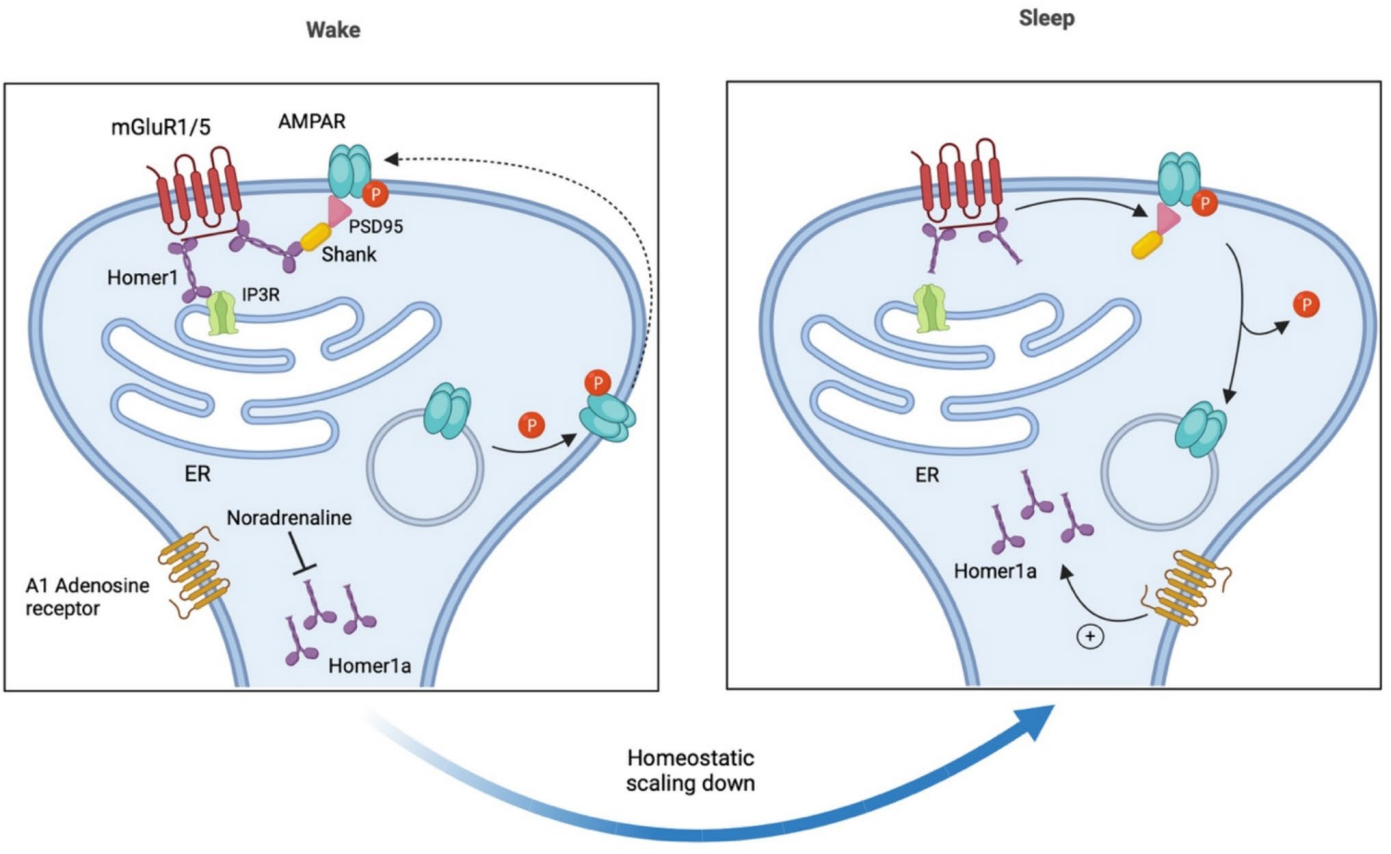
Switch between wake and sleep gated by Homer1a. During wakefulness, the long isoform of Homer1 links mGluR1/5 to shank scaffold proteins through its coiled-coil domain and to the C-terminal region of the IP3 receptor (IP3R) through its EVH1 domain. Homer1a targeting to the postsynaptic density during wakefulness is suppressed by noradrenaline. However, at the onset of sleep, noradrenaline levels decrease while adenosine levels rise. This increase in adenosine promotes Homer1a localization to the synapse, where Homer1a disrupts the mGluR1/5-Homer1-IP3R and mGluR1/5-Homer1-shank-PSD95-AMPA receptor (AMPAR) complexes. Additionally, Homer1a binding to mGluR1/5 facilitates agonist-independent signaling of mGluR1/5. PSD95, postsynaptic density protein 95; mGluR1/5, metabotropic glutamate receptor 1/5; ER, endoplasmic reticulum

Homer1a expression is also induced by sleep deprivation [256] and plays a role in homeostatic downscaling [255] as well as in synapse remodeling during sleep [257]. Homer1a protein shows reduced synaptic targeting during normal wakefulness but increased synaptic targeting during sleep or following sleep deprivation [257] (Fig. 7). This synaptic targeting is facilitated by elevated adenosine signaling through adenosine A1 receptors [257], which also promote Homer1a gene expression [258].

#### Homer1a in Synaptic Plasticity

Homer1a is involved in homeostatic downscaling, a non-Hebbian form of plasticity that regulates neuronal excitability by promoting the internalization of AMPA receptors from the synaptic surface [255, 257]. Homer1a knockout mice exhibit increased mEPSC amplitude and elevated synaptic surface levels of AMPA receptors, failing to display synaptic scaling [255]. This homeostatic downscaling occurs by disrupting the interaction between mGluRs and Shank within the mGluR-Homer1-Shank-PSD95-AMPA receptor complex, where the interaction between mGluRs and Shank is mediated by the long isoforms of Homer proteins [31]. Upon synaptic activation, the long Homer isoforms are replaced by Homer1a, which lacks the coiled-coil domain necessary for binding to the SH3 domain of Shank protein. This replacement disrupts the Shank interaction and averts the retention of GluA2-containing AMPA receptors at the synaptic surface. Additionally, in contrast to non-Hebbian forms of plasticity, Homer1a plays a role in suppressing Hebbian forms of plasticity. Overexpression of Homer1a abolished the maintenance of LTP in the hippocampal perforant path, which was restored after blocking Homer1a expression [259]. Furthermore, increased Homer1a expression in the hippocampus has been associated with impairments in LTP [260].

#### Homer1a in Memory

During exploration of a novel environment, both Homer1a and Arc are simultaneously induced in hippocampal neurons [252, 261, 262], suggesting that their coordinated activity may be essential for forming a memory related to that environment. As discussed in the “Arc” section, Arc, another immediate early gene, is critical for regulating synaptic plasticity and memory consolidation [252, 263, 264]. Spatial memory tasks also induce Homer1a expression [265], and its expression in the CA1 region is driven by novelty and correlates with memory encoding [266]. Homer1a is necessary for the formation and consolidation of fear memory [267–269]. Knockout mice of Homer1a show deficits in cued fear conditioning [32], and lower levels of Homer1a in aging mice correlate with cognitive deficits [270]. Conversely, overexpression of Homer1a impairs both fear conditioning [271] and spatial working memory [259]. Therefore, both lower and higher levels of Homer1a are detrimental to memory functions. Additionally, Homer1a contributes to extinction and extinction learning [272, 273].

#### Homer1a in Aging and Neurological Diseases

Reduced levels of Homer1a have been observed in aging, correlating with poor cognitive function [270, 274]. Psychotomimetic NMDA receptor antagonists, including ketamine and MK-801 [275, 276], as well as antipsychotics such as haloperidol and clozapine [276–279], also increase Homer1a expression. Altered Homer1a protein levels have been reported in patients with psychiatric disorders [280, 281]. In postmortem hippocampal CA1 from patients with schizophrenia, a decrease in long Homer1 isoforms and an increase in the short isoform Homer1a have been observed [282]. Acute cocaine administration in rodents induces a robust increase in Homer1a expression in the striatum, prefrontal cortex, and ventral tegmental area [283, 284]. Additionally, increased Homer1a expression has been shown to protect against NMDA-induced neuronal injury [285], attenuate stress responses [286], and promote stress resilience [260, 287]. Homer1a is also implicated in autism spectrum disorder [271, 288] and depression [258, 260].

### Arc

Activity-regulated cytoskeleton-associated protein (Arc), also known as activity-regulated gene 3.1 (Arg3.1), plays a key role in synaptic plasticity and memory [33, 289]. Arc is a synaptic protein and one of the effectors of BDNF, glutamate, dopamine, and serotonin signaling [162, 263, 290–292]. The Arc gene lacks homology with other genes or gene families and contains specific sequences typically found in retroviruses such as HIV [293, 294]. The Arc protein forms a virus-like particle that can encapsulate mRNAs and transfer them to neighboring neurons [295–298].

**Fig. 8:**
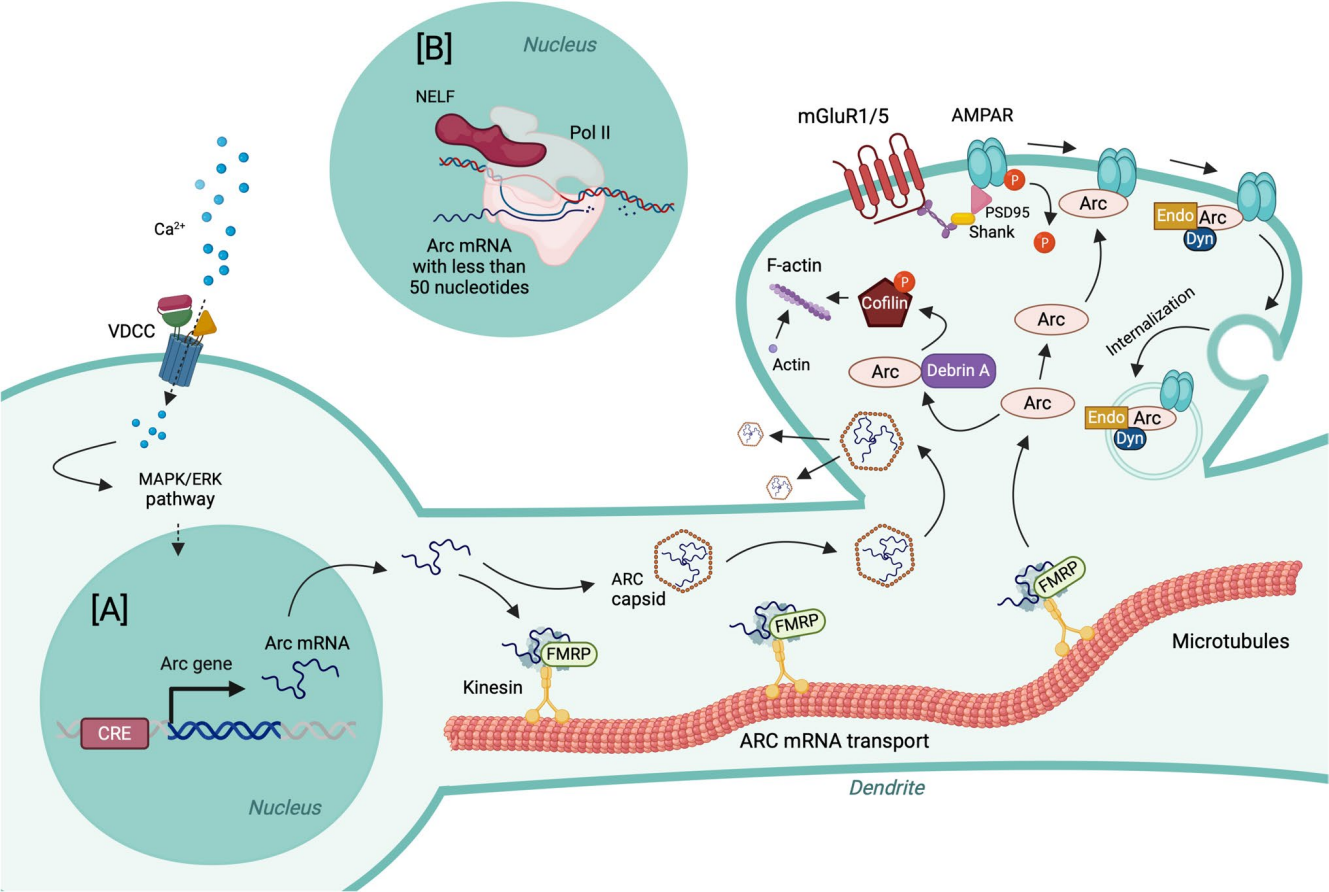
Arc role in synaptic functions. **A** Neuronal activation induces the rapid transcription of the Arc gene. Following transcription, cis-regulatory elements in the Arc mRNA facilitate its assembly into messenger ribonucleoprotein particles (mRNPs), which are then rapidly transported to dendrites via a kinesin/microtubule-dependent system. Additionally, Arc can self-assemble into capsids, enabling the transport of its own mRNA to dendrites and neighboring spines. At the synapse, Arc is involved in the formation of F-actin and the internalization of GluA1-containing AMPA receptors from the synaptic surface. **B** Neuronal activity also induces the transcription of a short form of Arc mRNA (less than 50 nucleotides) that regulates Arc protein translation at the synapse. NELF, negative elongation factor; Pol II, RNA polymerase II; VDCC, voltage-dependent calcium channel; Arc, activity-regulated cytoskeleton-associated protein; CRE, cAMP response element; FMRP, fragile X mental retardation protein; Endo, endophilin; Dyn, dynamin; F-actin, filamentous actin; mGluR1/5, metabotropic glutamate receptor 1/5; AMPAR, AMPA receptor; PSD95, postsynaptic density protein 95

Neuronal stimulation induces rapid transcription of the Arc gene and translocation of its mRNA from the nucleus to the dendrites [299, 300]. The transcription of the Arc gene is triggered by Ca^2^⁺ influx into the neuron [301]. Exposure to a novel environment induces Arc transcription in hippocampal CA1 neurons within 30 s [302], and translation of Arc mRNA, along with the accumulation of Arc protein in dendrites, occurs within 2 min [303–305]. After transcription, Arc mRNA binds to ribonucleoprotein, forming a complex that is rapidly transported to dendrites via a kinesin/microtubule-dependent mechanism [306, 307] (Fig. 8). In parallel, Arc can self-assemble into capsids, enabling the transport of its own mRNA to dendrites and neighboring spines [298, 308]. Arc plays a critical role in the formation of F-actin and internalization of GluA1-containing AMPA receptors from synaptic surface (Fig. 8).

#### Arc in Synaptic Plasticity

Arc facilitates the internalization of AMPA receptors from the synaptic surface [263] and plays a crucial role in both Hebbian (LTP and LTD) and non-Hebbian (homeostatic) plasticity [264, 308–311]. Inhibition of Arc expression impairs the maintenance of LTP [312], while sustained Arc expression promotes LTP consolidation [313]. In contrast, Arc knockout has been shown to produce no deficit in LTP in the CA1 area of the hippocampus [99], suggesting that alternative pathways may substitute for Arc function in LTP maintenance. Furthermore, Arc knock-in mice with mutated ubiquitination sites exhibit increased LTD, decreased theta burst stimulation-evoked LTP, and suppression of Group 1 mGluR priming of LTP [314]. Recent findings show that LTP induction in the hippocampus increases Arc-Arc dimer expression, which persists during the maintenance phase of LTP [315].

In addition to LTP, Arc is also essential for mGluR-dependent LTD [304]. Dendritic translation of Arc underlies the priming of mGluR-dependent LTD [316], and knockdown of Arc or blockade of Arc synthesis prevents mGluR-dependent LTD and AMPA receptor endocytosis [305]. Furthermore, deletion of Arc blocks the late phase of LTD [317]. Additionally, Arc mediates homeostatic synaptic scaling of AMPA receptor [310] and synapse-specific homeostatic plasticity [318, 319]. Therefore, Arc expression is critical for both LTP and LTD, and its inhibition impairs both.

#### Arc in Memory

Arc is required for the consolidation and reconsolidation of fear memory [320–322]. Disruption of Arc expression impairs the consolidation of long-term spatial memory [312] and inhibitory avoidance memory [323]. Arc-deficient mice display impairments in long-term spatial, fear, and recognition memory, while short-term memory remains unaffected [264]. Additionally, intrahippocampal infusion of Arc antisense oligodeoxynucleotides prior to training impairs the formation of long-term spatial and fear memories [312, 320, 324]. Fear extinction training increases the number of Arc-positive neurons in the basolateral amygdala; however, infusion of Arc antisense oligodeoxynucleotides into this region impairs long-term, but not short-term, fear extinction memory [34]. Furthermore, knockdown of Arc in the lateral amygdala impairs fear memory reconsolidation [321]. Optogenetic inhibition of hippocampal neurons expressing Arc during the acquisition of contextual fear memory suppresses memory retrieval [325].

#### Arc in Aging and Neurological Diseases

Normal aging causes behavioral experience-dependent decrease in Arc transcription as well as expression in the hippocampus [326–328], and this decrease in Arc transcription is associated with the increase in methylation of Arc gene [327]. Arc is also implicated in several neurological diseases, including Alzheimer’s disease [329–332] and schizophrenia [290, 333, 334]. Aβ causes sustained Arc expression, leading to the accumulation of Arc proteins in dendrites [335], and Arc overexpression leads to increased Aβ production [330]. Thus, Arc may enforce a positive feedback mechanism in amyloidogenesis [301]. Additionally, some genetic variants of Arc are considered a risk factor for schizophrenia, increasing the risk of developing schizophrenia [333, 336]. Mutations in Arc promotor and exon regions and hypermethylation in Arc promotor region have been shown to reduce the Arc expression in schizophrenia patients [334]. Arc expression is also altered in both animal models of depression and patients with major depressive disorder [337, 338]. Altered Arc expression is associated with seizures [339] and epilepsy [340], and it contributes to neuronal vulnerability to ischemia [341]. Moreover, Arc regulates anxiety behavior [342].

### Brain-Derived Neurotrophic Factor

**Fig. 9:**
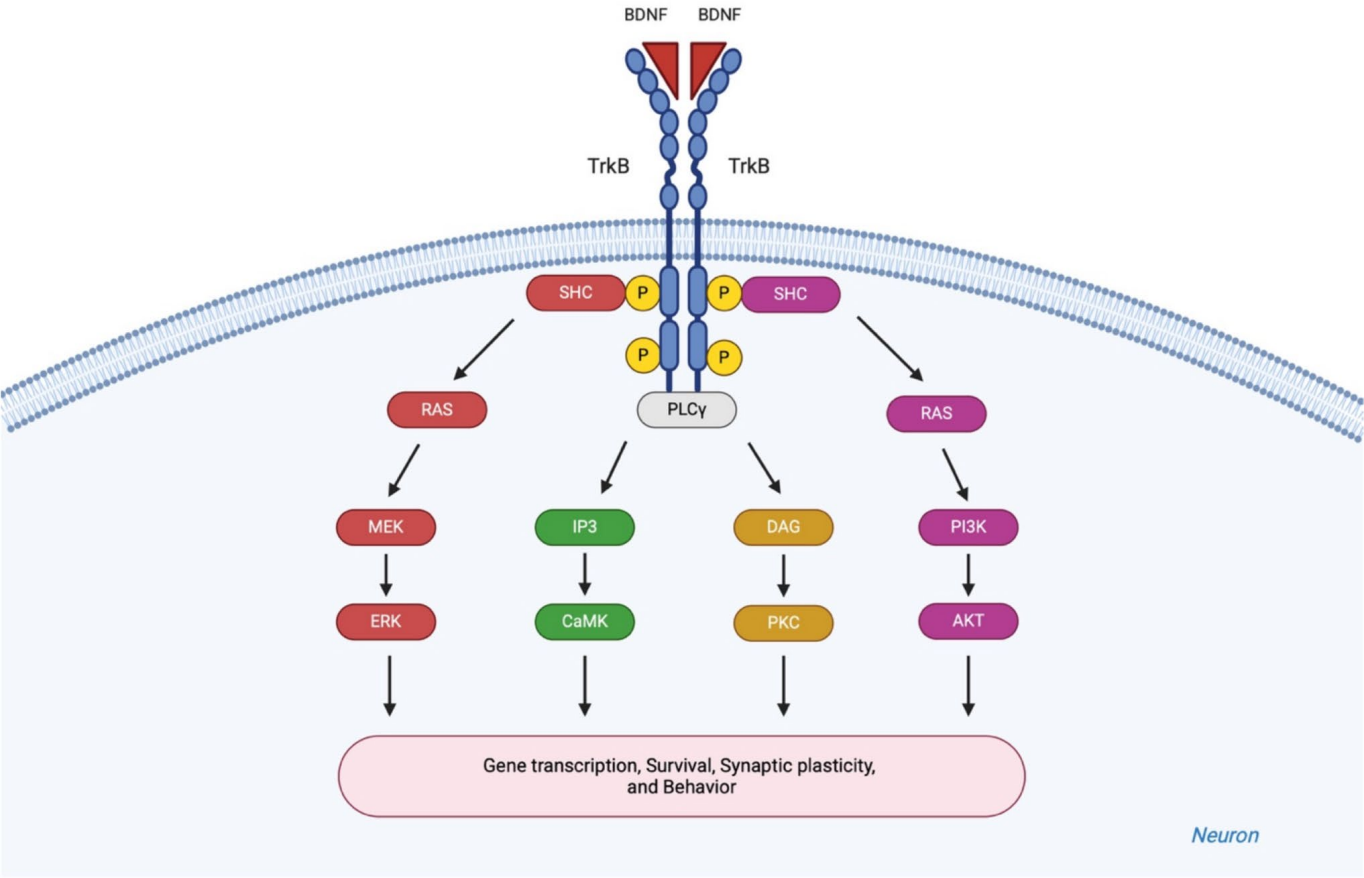
BDNF-mediated signaling pathways. BDNF binds to the extracellular domain of **TrkB**, forming homodimers that activate downstream intracellular signaling cascades, which include the Ras/MAPK, PLCγ, and PI3K/AKT pathways. These pathways are involved in various cellular responses such as growth, survival, differentiation, and synaptic plasticity. SHC, Src homology 2 domain; Ras, rat sarcoma; MEK, mitogen-activated protein kinase kinase; ERK, extracellular signal-regulated kinase; PLCγ, phospholipase C gamma; IP3, inositol triphosphate; CaMK, Ca2 +/calmodulin-dependent kinase; DAG, diacyl glycerol; PKC, protein kinase C; PI3K, phosphatidylinositol 3-kinase; AKT, protein kinase B

BDNF is a neurotrophin known for its roles in neuronal survival, axonal and dendritic growth and branching, neurotransmission, synaptic plasticity, and learning and memory [343]. BDNF is a 119 amino acids polypeptide initially synthesized as a 247 amino acids precursor protein called proBDNF, which includes an 18 amino acids signal sequence, a 110 amino acids prosequence, and a 119 amino acid mature segment [344]. Proteolytic cleavage of proBDNF by plasmin generates mature BDNF [53]. Both BDNF and proBDNF act through distinct receptor systems: proBDNF preferentially binds to the p75 neurotrophin receptor (p75NTR)—a common receptor for all neurotrophins—facilitating LTD and cell death under pathological conditions such as neurodegeneration, injury, or stress [345, 346]. In contrast, mature BDNF specifically binds to the tropomyosin receptor kinase B (TrkB), promoting LTP and cell survival [346–348]. BDNF binding induces receptors dimerization and activates multiple signaling pathways [343] (Fig. 9).

#### BDNF in Synaptic Plasticity

BDNF enhances LTP in the hippocampus and other brain areas [349–352]. This enhancement of LTP is mediated through adenosine A2A receptors [353]. Exogenous BDNF application to hippocampal brain slices has been shown to augment LTP, while a reduction in endogenous BDNF results in a decrease in LTP [350]. Heterozygous BDNF-knockout mice that express reduced levels of BDNF exhibit profound deficits in hippocampal LTP [343, 354], and this deficit can be rescued by recombinant BDNF expression [355, 356]. Additionally, BDNF induces late-phase LTP [357, 358], and this late-phase LTP has been shown to be both protein synthesis-dependent and independent [359]. Furthermore, disrupting activity-dependent BDNF expression impairs late-phase LTP [360]. In contrast to LTP, BDNF attenuates LTD in the hippocampus through the activation of phospholipase C [361] and the regulation of adenosine A2A receptors [362]. Moreover, BDNF modulates LTD in the visual cortex [363, 364] and prevents the induction of LTD in the developing visual cortex [365].

In addition to its role in Hebbian plasticity, BDNF signaling is essential for the non-Hebbian, homeostatic form of synaptic plasticity [35, 366, 367]. BDNF regulates the homeostatic scaling of both excitatory and GABAergic synapse strength [368, 369]. Furthermore, blocking spontaneous inhibitory events leads to downscaling of excitatory synaptic strength, which is dependent on both BDNF transcription and signaling [370], and BDNF contributes to homeostatic scaling by modulating the synaptic surface incorporation of AMPA receptors [371].

#### BDNF in Memory

BDNF levels are upregulated after learning [372] and during memory formation [373], and endogenous BDNF is essential for the formation and storage of long-term memory [36, 374]. In addition, the differential regulation of BDNF transcripts containing exons I, IV, and VI has been specifically associated with the consolidation of long-term memory [375–378]. During memory processing, different signaling pathways are involved in distinct brain areas [379], suggesting that BDNF exerts its functions through multiple signaling pathways that may be specific to each area. Exogenous BDNF treatment facilitates the consolidation of memory [380], while reduced expression of BDNF in both humans and animals causes deficits in episodic and declarative memory [381–383]. Clinical studies have shown that memory impairments in patients are associated with the BDNF Val66Met polymorphism, a condition where a methionine (Met) is substituted for valine (Val) at residue 66 of the BDNF protein, leading to reduced BDNF availability [382, 384]. Conversely, patients with BDNF haploinsufficiency—a condition where only one functional copy of the BDNF gene is present, resulting in reduced BDNF levels—show deficiencies in working memory [385]. Furthermore, heterozygous BDNF-knockout mice exhibit deficits in fear learning and fear extinction memory [37, 386], spatial reference memory [387], and object recognition memory [388]. Additionally, selective BDNF deletion in the hippocampus impairs both object recognition and spatial memory [389].

#### BDNF in Aging and Neurological Diseases

Aging suppresses chromatin activity at BDNF promoters [390], while BDNF gene transfer has been shown to promote healthy aging in mice [391]. A decline in BDNF-TrkB signaling during aging promotes microglial activation, which can be reversed by upregulating BDNF signaling [392]. Heterozygous BDNF-knockout mice, which have reduced BDNF expression, exhibit early-life learning deficits [393]. During aging, BDNF-mediated induction of LTP is impaired in the dentate gyrus [394]. Furthermore, aged individuals with the BDNF val66met polymorphism, where BDNF availability is restricted, show an early onset of memory deficits [395]. Similarly to aging, individuals with Alzheimer’s disease exhibit decreased BDNF mRNA levels in the hippocampus [396, 397], and reductions in BDNF exacerbate Alzheimer’s disease-related memory dysfunctions [398]. Increased BDNF levels and BDNF-mediated hippocampal neurogenesis prevent memory deficits in Alzheimer’s disease mouse models [399, 400]. Moreover, Tau downregulates BDNF in Alzheimer’s disease mouse models [401], while Alzheimer’s patients carrying the BDNF val66met polymorphism show increased Tau levels and Tau phosphorylation [402]. Additionally, exposure to stress, both maternal and early in life, alters BDNF methylation patterns [403–405]. The molecular chaperone FK506-binding protein (FKBP51) plays a role in the epigenetic regulation of the BDNF gene, contributing to stress and depression [406]. FKBP51 interacts with cyclin-dependent kinase 5 (CDK5), a kinase that activates DNA methyltransferase 1 (DNMT1), inducing methylation of the BDNF gene and repressing its expression. BDNF expression reduces stress and anxiety-related behavior [407] and restores synaptic plasticity and cognitive functions in conditions such as ischemia [408], Huntington’s disease [409], and fragile X syndrome [410].

### Neuronal Activity-Regulated Pentraxin

**Fig. 10:**
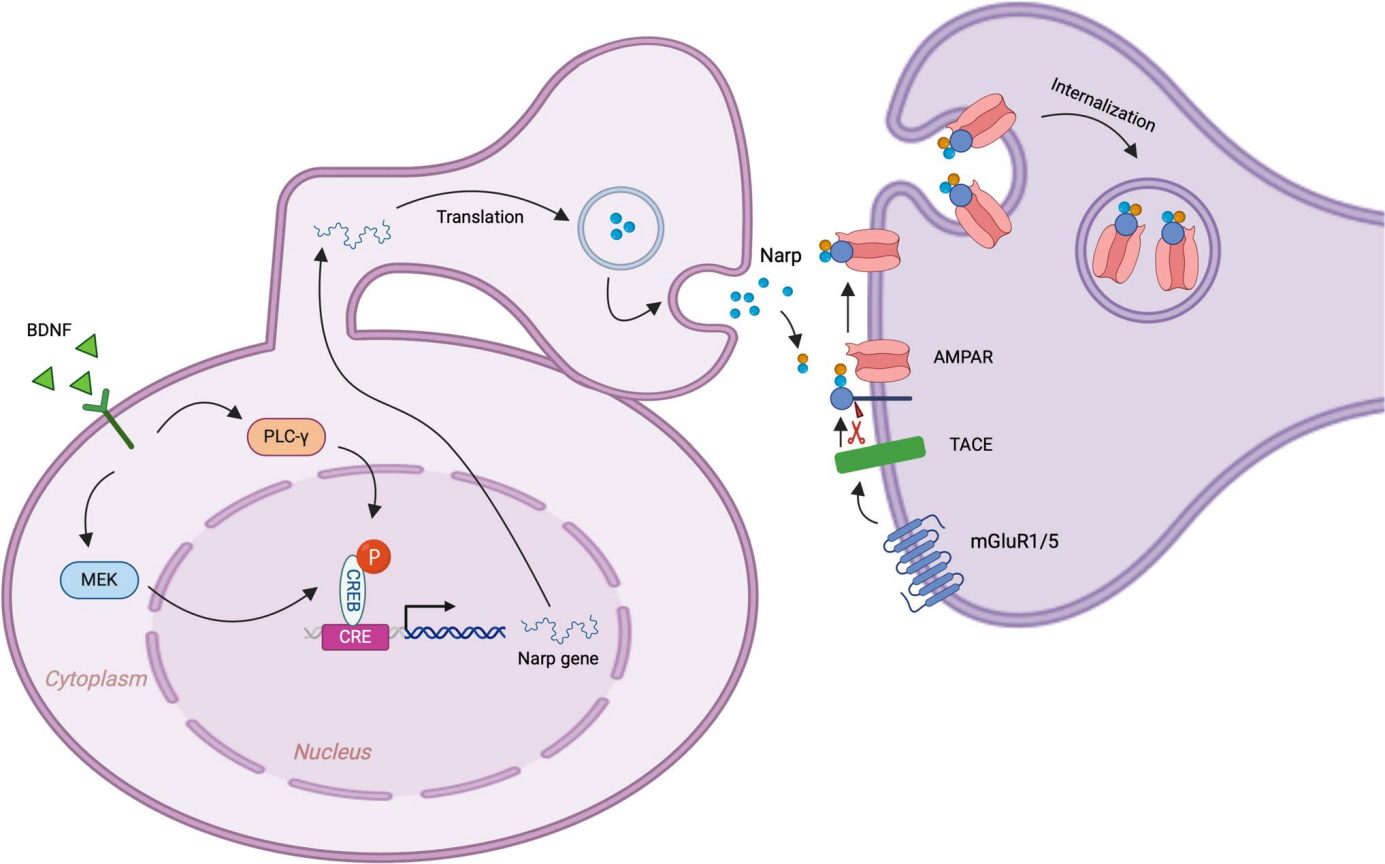
Narp-mediated AMPA receptor aggregation. Neuronal activity induced by BDNF activates the mitogen-activated protein kinase kinase (MEK) and phospholipase C gamma (PLCγ) pathways, leading to the phosphorylation of the transcription factors, such as CREB, and the promotion of Narp gene transcription. After translation of Narp mRNA in the cytoplasm, the Narp protein is delivered to the postsynaptic membrane, possibly facilitated by perineuronal nets. At the postsynaptic membrane, Narp forms a complex with other pentraxins and binds to the pentraxin receptor. In the presence of activated mGluR1/5, tumor necrosis factor-alpha converting enzyme (TACE) cleaves the transmembrane domain of the pentraxin receptor. This cleavage allows the internalization of the pentraxin complex and associated AMPA receptors through endocytosis. BDNF, brain-derived neurotrophic factor; CRE, cAMP response element; AMPAR, AMPA receptor; mGluR1/5, metabotropic glutamate receptor 1/5

Neuronal activity-regulated pentraxin (Narp) is a secreted, self-multimerizing protein expressed in neurons, and its expression is rapidly upregulated in response to increased synaptic activity [44, 411]. Narp is localized to synaptic compartments and, upon activation, forms protein clusters that are covalently linked through disulfide bonds by interacting with other Narp molecules and related pentraxins [412]. These clusters bind to AMPA receptors, creating aggregates of AMPA receptor-Narp complexes at excitatory synapses [411] (Fig. 10). This clustering of AMPA receptors facilitates synaptogenesis at excitatory spines [412]. Deletion of Narp decreases the number of excitatory synaptic inputs onto parvalbumin-expressing inhibitory neurons and reduces the net excitatory synaptic drive [413]. Therefore, the lack of Narp suppresses the inhibitory component essential for synaptic plasticity. Notably, Narp function is dependent on BDNF. Studies have shown that downregulation of BDNF decreases Narp expression, while BDNF enhances Narp transcription [414].

#### Narp in Synaptic Plasticity

Narp regulates the homeostatic scaling of excitatory synapses on parvalbumin-expressing interneurons [415]. Specifically, Narp accumulates at excitatory synapses that form contacts with parvalbumin-expressing interneurons. An upregulation in network activity leads to a homeostatic increase in the strength of excitatory inputs onto these interneurons, thereby enhancing inhibitory drive. However, this homeostatic increase in excitatory strength is absent in Narp knockout mice [415]. Moreover, Narp deletion not only decreases the number of excitatory synaptic inputs onto parvalbumin-expressing interneurons but also reduces their net excitatory synaptic drive, thereby preventing Narp knockout mice from exhibiting ocular dominance plasticity [413]. Additionally, Narp is upregulated during BDNF-induced LTP in the dentate gyrus [416] and is crucial for BDNF-mediated glutamatergic transmission and synaptic plasticity in the hippocampal mossy fiber pathway [414].

#### Narp in Memory and Behavior

Object-place recognition learning increases Narp expression in the dentate gyrus of rats [417]. Impairments in memory consolidation are associated with reduced Narp transcription in the hippocampus, driven by increased DNA methylation and decreased histone acetylation in its promoter region [418]. Furthermore, a decrease in Narp has been shown to be involved in social memory deficits in mice that underwent juvenile social isolation [419]. Narp knockout mice show impairments in extinction of morphine conditioned place preference [420], cognitive inflexibility, and deficits in using the current value of a reward to guide behavior, a critical brain function in decision-making and behavior control [46, 421]. Additionally, localized disruption of endogenous Narp trafficking to axons in the prefrontal cortex of mice blocked reinforcer devaluation behavior [422]. Therefore, Narp seems to play an essential role in information processing required for appropriate devaluation performance.

#### Narp in Addiction and Neurological Diseases

Acute administration of methamphetamine increases Narp mRNA levels in the prefrontal cortex, while opiate and morphine withdrawal induce Narp expression in the extended amygdala [423, 424]. Narp modulates aversive responses to morphine withdrawal [424], and Narp in the medial prefrontal cortex is critical for the extinction of morphine-conditioned place preference [420]. In contrast, amphetamine and cocaine treatment do not increase Narp expression, except in the amygdala when very high doses of amphetamine are administered [425]. Narp knockout mice exhibit normal locomotor sensitization and conditioned place preference but show marked resistance to the extinction of morphine place preference [45]. These observations suggest that Narp may regulate plasticity processes underlying drug addiction.

Narp is also implicated in neurological diseases. Seizures induced by electroconvulsive stimulation, a commonly used antidepressant treatment, cause sustained Narp expression in the hippocampus [426], and Narp mediates the antidepressant effects of electroconvulsive seizures [427]. Furthermore, BDNF signaling-mediated Narp upregulation is essential for antidepressant effects in mice [428], and Narp knockout mice fail to exhibit this antidepressant effect [427]. Beyond its role in antidepressant effects, Narp contributes to the development of L-DOPA-induced dyskinesia in Parkinson’s disease [429] and is implicated in autism spectrum disorder [419]. Additionally, Narp mRNA levels in the dorsolateral prefrontal cortex of patients with schizophrenia, bipolar disorder, and major depressive disorder have been shown to be reduced [430]. These levels were positively correlated with GAD65, an enzyme responsible for GABA synthesis, suggesting that reduced excitatory drive due to lower Narp levels may lead to decreased GABA synthesis and reduced inhibitory capacity in these interneurons.

## Concluding Remarks

In addition to the IEGs described here, numerous other IEGs associated with memory functions and synaptic plasticity have been reported (see Table 1). While their roles in these processes are becoming clearer, the literature on them remains limited. Nevertheless, our review of some of these IEGs suggests that they play fundamental roles in behavioral learning and in the formation and consolidation of various types of episodic memory. They are also crucial for synaptic and structural plasticity. The function of these IEGs is altered during aging and in brain diseases. Recent studies indicate that, during learning, IEG-positive neurons encode and store information required for memory recall, suggesting their involvement in the formation of memory traces. These IEG-positive neurons are distributed across brain regions within interconnected neuronal ensembles, and disruption of these ensembles results in memory recall deficits. Despite advances in understanding the role of IEGs in learning, memory, and synaptic plasticity, the precise mechanisms underlying their involvement in these processes remain unclear. Additionally, the AP-1 dimer composed of c-Fos and c-Jun is one of the most studied examples within this family of transcription factors. As described in Fig. 1, numerous dimer combinations are possible; however, the functions of most remain unknown. While all the IEGs reviewed here are implicated in memory processing, formation, and synaptic plasticity, Npas4 has been shown to play a specific role in inhibitory synapse formation. In contrast, within the group of IEGs encoding effector proteins, Zif268, Arc, and Narp appear to play direct roles in the internalization of synaptic surface AMPA receptors—a critical step in synaptic depression and homeostatic downscaling—whereas BDNF is involved in both Hebbian and non-Hebbian forms of synaptic plasticity. Furthermore, dysfunction or alterations in these IEGs are frequently associated with aging and various neurological and psychiatric diseases, and Npas4, Zif268, and Narp have been implicated in addiction and addictive behaviors. Continued research on IEGs is essential for understanding how these genes coordinate and regulate transcription, participate in different forms of synaptic plasticity, and promote the formation of memory traces and the consolidation of long-term memories. Such research is also critical for identifying therapeutic targets to address memory deficits and neurological diseases.

## Data Availability

No datasets were generated or analysed during the current study.
